# Viral interferon antagonism shapes host tropism

**DOI:** 10.1371/journal.ppat.1013544

**Published:** 2025-10-07

**Authors:** Felix Streicher, Amandine Chantharath, Nolwenn Jouvenet

**Affiliations:** 1 Virus Sensing and Signaling Unit, Institut Pasteur Paris, Université de Paris, CNRS UMR 3569, Paris, France; 2 Institute of Virology, Charité-Universitätsmedizin Berlin, Corporate Member of Freie Universität Berlin, Humboldt-Universität zu Berlin, Berlin, Germany; 3 Centre International de Recherche en Infectiologie (CIRI), Inserm U1111, CNRS UMR5308, Ecole Normale Supérieure de Lyon, Université Claude Bernard, Lyon, France; Mount Sinai School of Medicine, UNITED STATES OF AMERICA

## Abstract

Outbreaks of zoonotic viruses in human populations highlight the need to understand the molecular factors that influence viral host tropism and interspecies transmission. A virus’s host range is determined not only by the expression of host factors that facilitate viral entry and replication, but also by the virus’s ability to evade host antiviral immunity. The interferon (IFN) response is a potent immune defense rapidly mobilized upon viral infections. To overcome this immune barrier and establish infection, viruses continuously evolve evasion strategies. This review illustrates, through a series of examples, how species-specific interactions between viruses and the IFN response erect interspecies barriers. We discuss the necessity to develop cellular models derived from viral reservoir hosts, as well as machine learning approaches, to better grasp species-specific viral sensitivity to the IFN response. These approaches will be essential for understanding viral interspecies transmission and guiding effective pandemic preparedness measures.

## Introduction

Cross-species transmission (CST), where a virus is transmitted from one animal species to another, can have significant health impacts on humans, livestock, and wildlife [1–3]. Viral zoonoses such as Spanish flu and AIDS, caused, respectively, by an H1N1 subtype of the influenza A virus (IAV) and HIV-1, have profoundly influenced human history. More recent examples include SARS-CoV-2 and Mpox pandemics, as well as the ongoing spillover of an avian-borne H5N1 influenza to numerous species of mammals [4–6]. CST of viruses is a complex process influenced by environmental and biological factors, such as unprecedented contact between natural and novel hosts due to human activities like habitat expansion and intensified agricultural practices [1]. The bat-borne virus Nipah, for instance, emerged in Malaysia in 1998 following the expansion of pig farming, leading to pig-to-pig and pig-to-human transmission via aerosol [7].

Upon contact with the new host, the virus must pass physical barriers such as the skin, mucus, or stomach acids of the novel host to reach host cells. The virus must then encounter favorable molecular conditions, including the expression of host factors allowing viral entry, replication, and spread. Additionally, viruses must also cope with the host antiviral systems, such as apoptosis and immune defense.

**Fig 1:**
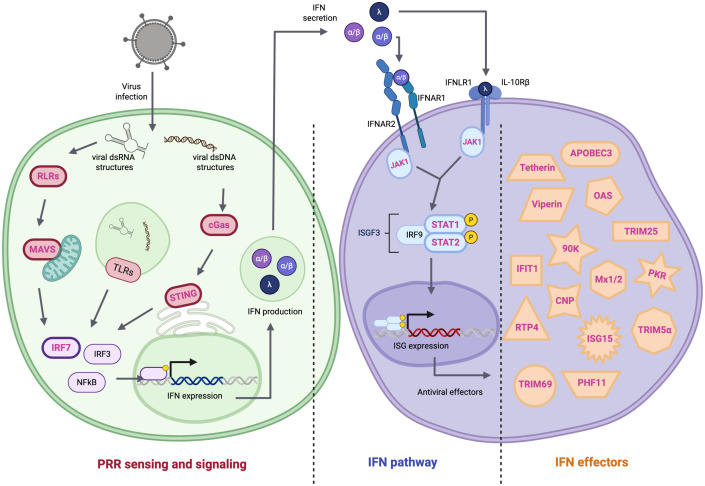
IFN-dependent antiviral response. The innate immune response is conserved across mammalian species, but it lacks memory capabilities and needs fine-tuned regulatory mechanisms to avoid causing immunopathology. Upon entry into cells, viral components are detected by germline-encoded proteins called pattern recognition receptors (PRRs). These include membrane-bound toll-like-receptors (TLRs) and cytosolic sensors such as RIG-I-like receptors (RLRs) and cGAS. They are activated depending on the nature and cellular localization of viral antigens. Once activated by the interaction with viral components, PRRs initiate signaling pathways that lead to the production and secretion of type I and III interferons (IFN). Secreted IFNs bind to their respective receptor complexes (IFNAR1/2 or IFNLR1/IL-10Rβ), which trigger the JAK-STAT pathway. This leads to the activation of STAT1 and STAT2, which associate with IRF9 to form the ISGF3 transcription factor complex, inducing the expression of hundreds of IFN-stimulated genes (ISGs), establishing a robust antiviral state. Proteins that contribute to interspecies transmission barriers for viruses are outlined by a bold line and written in pink, and described in the text. Created in BioRender. https://urldefense.com/v3/__https://BioRender.com/ai0cajm__;!!JFdNOqOXpB6UZW0!qE_dfBruIlmSU-rmo3ya4I37cy0eQrZ8nhz6vRSpTfifiY_79LhLogYEkPhs4-N9D11wwYm62NAE8nJ7CU4_ZdAFDg$.

The innate immune response, which is at the heart of the antiviral response, is a rapid, potent, and nonspecific response. It is initiated when host proteins recognize viral components, triggering signaling cascades that lead to the expression of cytokines and effectors that inhibit viral replication (Fig 1). In the absence of viral countermeasures, the innate immune response rapidly controls viral replication. Consequently, viruses have evolved effective strategies to evade this response. A common strategy consists of expressing a viral protein that binds to a cellular protein crucial for the IFN response, inhibiting its activity by cleaving it, targeting it for degradation, or obstructing its interaction with another key IFN protein [8]. Orthologous proteins—those derived from an ancestral protein in different species—may resist viral evasion, thereby limiting viral propagation in novel nonnatural hosts.

In this review, we provide an overview of examples illustrating how the ability of viral proteins to overcome the mammalian IFN response affects CST and, therefore, the viral host range.

### Species-specific targeting of PRRs and downstream factors by viral proteins

The initiation of the IFN response relies on the identification of viral genomic material by pathogen recognition receptors (PRRs) [9]. Once bound to viral nucleic acids or proteins, PRRs adopt a novel conformation that enables their recognition by adaptor proteins. These protein complexes then recruit and activate kinases, which phosphorylate transcription factors such as IRF3/7 and NF-kB. The phosphorylated transcription factors translocate to the nucleus, ultimately inducing the expression of IFNs and other cytokines [10] (Fig 1). The examples below illustrate how viral evasion of this signaling cascade influences the viral host range.

#### RIG-I.

Retinoic acid-inducible gene I (RIG-I) is a PRR recognizing short (10–300 bp) viral dsRNA or stem-loop ssRNA molecules harboring 5′-triphosphate ends with regions enriched in poly-U/UC or AU sequences [11]. RIG-I also detects RNA molecules derived from viral DNAs. Herpes simplex virus 1 (HSV-1) is a DNA virus that naturally only infects humans [12]. In human THP1, HeLa, and 293T cells, RIG-I detects viral dsRNA that is generated during the replication of HSV-1. However, the UL37 deamidase expressed by HSV-1 inactivates human RIG-I (hRIG-I) by hydrolyzing the side chain of its asparagine residue N495. Deamidation of N495, which is exposed on the surface of the helicase domain of hRIG-I, renders it unable to bind RNA molecules, thus blocking its ability to trigger downstream host immune signaling [13]. Sequence alignments indicate that RIG-I of several nonhuman primates possess the residue N495 and are thus also likely to be deamidated by UL37 [14]. This is in line with several nonhuman primates being susceptible to HSV-1 infection [15]. 2-D gel electrophoresis performed in 293T cells stably expressing murine RIG-I (mRIG-I) or hRIG-I, and infected with either wild-type HSV-1 or a mutant virus expressing a deficient UL37, revealed that mRIG-I is resistant to deamidation [14]. This resistance resulted in a higher potency of mRIG-I in restricting HSV-1 infection in murine embryonic fibroblasts (MEFs) expressing either mRIG-I or hRIG-I. Replacing the human N495 by the mRIG-I lysine (hRIG-I-K495) resulted in the resistance to HSV-1-mediated deamidation [14]. RIG-I from other mammals also contains nonasparagine residues at position 495 that are not subjected to deamidation, suggesting a potentially diminished susceptibility to HSV-1 infection of these species [14].

#### TRIM25*.*

The ubiquitin ligase TRIM25, which activates RIG-I via ubiquitination, is targeted by the nonstructural protein 1 (NS1) of IAVs [16]. These viruses from the *Orthomyxoviridae* family are naturally maintained in ducks and wild waterfowl, but also circulate in humans, horses, dogs, and pigs [17]. NS1 of IAVs bind to the central coiled-coil (CCD) region of human TRIM25 (hTRIM25), blocking its oligomerization and thus its ability to induce ubiquitination of hRIG-I^16^. Co-immunoprecipitation (co-IP) studies performed in 293T cells showed that NS1 encoded by human, swine, and mouse-adapted IAVs interacted with hTRIM25 [18]. In contrast, *in vitro* binding assays revealed that none of these NS1 proteins interacted with murine TRIM25 (mTRIM25) [18]. Biochemical assays performed with chimeric human/mouse TRIM25 revealed that amino acids in the CCD region of the 2 species are responsible for their differential interaction with NS1. These studies suggest that NS1 encoded by human, swine, and mouse-adapted IAVs could overcome the IFN response in humans.

#### MAVS.

The recognition of viral RNA by RIG-I or MDA5 triggers the activation of the mitochondrial adaptor protein MAVS [19]. Hepatitis C (HCV) and A viruses (HAV), human pathogens from the *Flaviviridae* and *Picornaviridae* families, respectively, express proteases that cleave MAVS in a species-specific manner. HCV protease NS3/4A cleaves overexpressed MAVS from a dozen primate species, including humans and chimpanzee [20–22], in 293T cells. However, MAVS from other primate species, such as olive baboon, rhesus macaque, spider monkey, and dusky titi monkey, remain functional despite the presence of NS3/4A [22]. Sequence comparisons of MAVS from different primate species identified residue 506 as key for NS3/4A cleavage. An ancestral MAVS-sensitive valine at this position evolved into MAVS-resistant glycine and alanine in rhesus macaque and spider monkey, respectively, potentially due to the selective pressure of an infection by an ancient HCV-like virus. HCV and other hepaciviruses have never been found in primate species expressing these ‘resistant’ MAVS, underscoring the importance of MAVS and the IFN response in determining the outcome of viral infection in closely related species [22]. Similar to HCV NS3/4A, the HAV protease 3ABC cleaves hMAVS but not mouse MAVS in murine *Mavs*^−/−^ embryonic fibroblasts co-transfected with either murine or hMAVS and 3ABC [23]. Thus, the ability of HCV and HAV to evade MAVS-mediated IFN responses likely plays a role in defining their host species range.

#### cGAS.

The primary sensor of foreign or cytoplasmic host DNA in human cells is the cyclic GMP-AMP synthase (cGAS) [24]. This nucleotidyltransferase catalyzes the production of cyclic GMP-AMP (cGAMP) from GTP and ATP upon binding to cytosolic dsDNA molecules. Binding is sequence-independent and its efficiency increases with the length of the DNA molecule, favoring longer sequences [25]. In addition to targeting hRIG-I, tHSV-1 UL37 also deamidates human cGAS (hcGAS) [26]. 2-D gel electrophoresis and tandem mass spectrometry analysis performed in 293T cells expressing UL37 and hcGAS showed that the viral protein deamidates hcGAS at residue N210, which is key for catalyzing cGAMP synthesis and activating downstream signaling [26]. This N210 residue is conserved in human, gorilla, and mouse cGAS, but not in other nonhuman primate orthologs [26]. Complementing mouse L929 cells with cGAS from four primate species that encoded amino acids other than N at position 210 showed that these cGASs more potently reduced HSV-1 replication than human or gorilla cGAS. These studies illustrate how a single residue in a cellular sensor can be determinant for controlling viral replication.

#### STING.

Human STING (hSTING), a crucial adaptor protein activated by cGAS, is one of the substrates for the NS2B3 protease complex of several orthoflaviviruses. These include the proteases of the four serotypes of dengue virus (DENV), Zika virus (ZIKV), and West Nile virus (WNV), all of which are pathogenic in humans, as well as Duck Tembusu virus (DTMUV), which is highly pathogenic in ducks [27–30]. The ability of orthoflavivirus NS2B3 to cleave hSTING has been demonstrated in human 293T and A549 cells, as well as in golden hamster BHK-21 cells [27,28,30]. Additionally, hSTING cleavage also occurred in primary monocyte-derived dendritic cells infected with DENV-2 [27] and in human dermal fibroblasts infected with ZIKV [29]. DTMUV NS2B3 cleaves duck and human but not mouse STING in golden hamster BHK-21 cells transfected with DTMUV NS2B3 and STING orthologs [30]. Similarly, DENV and ZIKV NS2B3 are unable to cleave mSTING in 293T cells expressing NS2B3 and STING orthologs [27,29,31], consistent with the inability of DENV and ZIKV to replicate in mice. Biochemical and mutational studies in 293T cells have revealed that an ‘RG’ motif at residues 78 and 79 of hSTING is necessary and sufficient for cleavage by the proteases of ZIKV and DENV [29,31]. Further co-transfection assays showed that STING orthologs from chimpanzee, rhesus, and squirrel monkeys are also resistant to ZIKV NS2B3 cleavage [29], while STING orthologs from chimpanzee, rhesus macaque, and common marmoset are resistant to DENV NS2B3 cleavage [31]. Using a database with genetic information of over 5,000 mammals, the RG motif that is sensitive to NS2B3 cleavage was found only in STING proteins of three apes (gorillas, orangutans, and gibbons) and three rodents (chinchillas, naked mole rats, and desert woodrats) [31]. The ability of orthoflavivirus NS2B3 to cleave STING thus contributes to delineating viral host range.

#### IRF7.

IRF7 is a key transcription factor of the transcriptional activation of IFNs, is targeted by the nucleocapsid (N) protein of porcine deltacoronavirus (PDCoV), a well-established IFN signaling antagonist, in porcine PK-15 and human 293T cells [32]. Mass-spectrometry analysis in porcine LLC-PK1 cells expressing a tagged version of N identified porcine IRF7 (poIRF7) as a cellular partner [32]. Co-IP assays in LLC-PK1 cells validated the interaction between N and poIRF7 [32]. Further co-IPs assays in 293T cells showed that N co-immunoprecipitated with poIRF7, but not with human IRF7 (hIRF7) nor chicken IRF7 (chIRF7). Biochemical and mutational assays showed that N promoted poIRF7 proteasome-mediated degradation. Therefore, the species-specific binding of PDCoV N to poIRF7 contributes to the inhibition of the IFN induction pathway in its natural host, the pig.

#### PKR.

Protein kinase R (PKR) is an IFN-inducible serine-threonine kinase that becomes activated upon stress or viral infection. PKR self-activates through autophosphorylation and dimerization upon detecting long dsRNA that is produced during the replication of all RNA viruses [33]. This activation leads to the phosphorylation of alpha subunit of eukaryotic translation initiation factor 2alpha (eIF2α), blocking translation initiation, including that of viral mRNA. This inhibition triggers apoptosis of infected cells, limiting the spread of infection. Due to its potent antiviral activity, many viruses of diverse families have evolved PKR antagonists [33].

For instance, the replication of mouse adenovirus type 1 (MAV-1), a DNA virus that belongs to the *Adenoviridae* family, triggers the degradation of murine PKR (mPKR) through proteasome-dependent degradation in several mouse cell lines [34]. Transfections of human and mouse cells with plasmids expressing mPKR and MAV-1 E4orf6, a ubiquitin-ligase, showed that E4orf6 induces mPKR degradation [35]. Experiments performed in MEFs knocked out for PKR expression and stably expressing mPKR or hPKR showed that hPKR, by contrast to mPKR, was not degraded by MAV-1 infection [35]. hPKR and mPKR share only 56% identity and 69% similarity, which could explain why MAV-1 infection does not induce degradation of hPKR.

Like other herpesviruses, human cytomegalovirus (HCMV) exhibits a narrow host range, due to long-term co-evolution with its host [36]. Consistent with this, the HCMV PKR antagonist TRS1 (hTRS1) inhibits only hPKR and not PKR orthologs of Old World monkeys [37]. Unexpectedly, in infection assays, TRS1 from CMVs infecting squirrel monkeys (SmTRS1) exhibited the ability to inhibit hPKR, while other TRS1 orthologs from primate CMVs failed to exert this inhibitory effect on hPKR [38]. The differential inhibition of hPKR by these TRS1 orthologs is facilitated by a unique amino acid residue located within PKR at position 489. Mutating this residue in hPKR by the residue encoded by African Green Monkey (F489S) resulted in a PKR that is resistant to inhibition by hTRS1, although it remained susceptible to the inhibitory effects of SmTRS1 [38]. Despite the identification of this pivotal residue, the mechanisms by which nonhuman primate CMVs evade the IFN response in human cells remain unclear.

Poxviruses are a large family of dsDNA viruses that infect a large range of animals. The *Chordopoxvirinae* subfamily comprises 18 genera of viruses infecting vertebrates [39]. They enter cells *via* widely conserved receptor proteins [40]. Productive infection is thus mainly determined by their ability to antagonize their host immune response [41]. Vaccinia virus (VACV), which infects mainly cattle, encodes a dsRNA-binding protein E3 that shields viral dsRNA from PKR detection [42]. While a VACV lacking the *E3L* gene encoding for E3 protein was not able to replicate in wild-type human HeLa cells, stable knockdown of *PKR* expression in these cells rescued viral replication of the mutant virus [43]. Opposite to that, an E3L-lacking VACV mutant virus was able to replicate in Syrian hamster (*Mesocricetus auratus*) BHK-21 cells. While these differences were initially thought to be linked to different expression levels of PKR in human and hamster cells, later experiments revealed species-specific sensitivity of PKR to VACV E3 [44,45]. A HeLa-cell-based luciferase assay indeed showed that E3 inhibited human, mouse, Armenian, and Chinese hamster PKR, but not orthologs from Syrian and Turkish hamster species [45]. Follow-up mutational and domain-swapping experiments identified a region linking the dsRNA-binding domain to the kinase domain of PKR as responsible for the phenotype [45]. The specific mechanism of how this linker sequence influences PKR-antagonism, however, remains elusive.

Poxviruses, including VACV, also encode K3, which is a pseudosubstrate mimic of eIF2α [41] that binds to the kinase domain of PKR and thereby obstructs its interaction with eIF2α [44,46]. Reporter luciferase assays in which luciferase expression is reduced due to translational inhibition caused by PKR over-expression revealed that K3 inhibits PKR in a species-specific manner [47,48]. For instance, K3 from sheeppox and goatpox viruses efficiently inhibit sheep, goat, and human PKR, but only weakly inhibit cow and mouse PKR [47]. In general, K3 from a given poxvirus efficiently inhibits PKR from its natural hosts [48]. Human, pig, sheep, and Armenian hamster PKRs were resistant to VACV K3, whereas mouse, horse, camel, cow, and Syrian hamster PKRs were sensitive [45,48]. Mutational experiments performed with Armenian and Syrian hamster PKRs identified residues 463 and 464 as keys for K3 antagonism [45]. Another study investigating the sensitivity of PKR from diverse chiropteran species to VACV E3 using a surrogate yeast system and a luciferase assay in HeLa PKR knockout cells also revealed host species-specific differences [49]. Human PKR and orthologs from *Rhinolophus sinicius*, *Desmodus rotundus*, *Eptesicus fuscus*, *Myotis myotis*, and *Myotis velifer* were not antagonized by VACV E3, while PKRs from *Pteropus alecto*, *Noctilio albiventris*, *Natulus tumidirostris*, and *Molossus molossus* were sensitive to the viral protein [49]. Evolutionary-guided approaches identified various fast-evolving stretches of amino acid sequences in chiropteran PKR, which drive host-poxvirus K3 specificity and potentially influence species-transmission patterns [49]. However, the poxvirus host range cannot be predicted from the ability of their K3 orthologs to antagonize PKR. In fact, some K3 orthologs inhibit PKR from species that are not their natural hosts [48]. For instance, the camelpox K3 ortholog antagonizes all primate PKRs, with the exception of rhesus macaque PKR, despite primate species not being susceptible to the virus. The reverse is also true: lumpy skin disease virus and variola virus only weakly inhibit PKR from their natural hosts, cattle and humans, respectively. These two poxviruses are likely to rely on proteins other than K3 to evade PKR antiviral function in their natural host [41].

### Species-specific inhibition of the IFN signaling pathway by viral proteins

Secreted type I and type III IFNs interact with their respective receptor complexes on infected and surrounding cells [50] (Fig 1). These interactions induce the activation of the JAK kinase that phosphorylates STAT proteins. The activation of the pathway induces the expression of hundreds of IFN-stimulated genes (ISGs), some of which exhibit antiviral potential [51]. Viral proteins target STAT1 and STAT2 from their natural hosts to hamstring this antiviral response.

#### Jak1.

Several filoviruses cause severe hemorrhagic fevers in humans and nonhuman primates [52]. The subgenus *Orthomarburgvirus* comprises two distinct lineages of a single species: Marburg virus (MARV) and Ravn virus (RAVV) [52]. When expressed in stimulated human Huh7 cells, the matrix protein VP40 of both viruses inhibits STAT1 phosphorylation and Jak1 autophosphorylation [53], suggesting that VP40 suppresses Jak1 kinase activity. In contrast, VP40 from both strains fails to prevent STAT1 phosphorylation in murine Hepa1.6 cells [53]. A mouse-adapted strain of RAVV, differing from the parental virus by only 7 amino acids in VP40, was able to interfere with STAT phosphorylation in both human and murine cells [53]. Two amino acids in VP40 were identified as critical for the species-specific inhibition of IFN signaling [53]. The precise mechanism by which these residues are linked and modulate Jak1-driven phosphorylation remains to be investigated.

#### STAT1/2.

Simian virus 5 (SV5), a *Rubulavirus* that replicates efficiently in primates but poorly in mice, inhibits the activation of ISGs in human 2FTGH fibroblasts but not in murine BF cells [54]. The SV5 V protein mediates the proteasomal degradation of STAT1 in 2FTGH cells [55]. Experiments performed in STAT1-deficient human U3A cells showed that both murine and human STAT1 proteins are susceptible to degradation by the V protein [56]. This is unsurprising given that murine and human STAT1 orthologs share 92.4% amino acid identity. Therefore, the inability of SV5 to antagonize IFN signaling in murine cells is not linked to its interaction with STAT1. Instead, the species specificity of SV5 can be attributed to differences between human and mouse STAT2 proteins. Expression of V and hSTAT2 in murine NIH3T3 cells triggers the degradation mSTAT1 and thus the inhibition of IFN signaling [56]. Consistent with this, SV5 infectious titers were higher in NIH3T3 cells expressing hSTAT2 compared to control cells, indicating that hSTAT2 confers a growth advantage for SV5 in murine cells [56]. Yeast two-hybrid system and protein capture approaches showed that the V protein acts as an adaptor molecule, linking the ubiquitin-ligase complex DDB1/Cullin 4a to STAT2/STAT1 heterodimers, thereby triggering STAT1 ubiquitination and subsequent degradation [57]. Thus, STAT2 serves as a key determinant of SV5 host range restriction.

The V protein of the Nipah virus (NiV), a paramyxovirus circulating in *Pteropus* fruit bats, targets STAT1 and STAT2 proteins in human fibrosarcoma (2FTGH) cells [58]. Unlike the SV5 V protein, it does not induce the degradation of STAT1 in a STAT2-dependent manner. Instead, it forms high molecular weight complexes with both STAT1 and STAT2, preventing their nuclear translocation [58]. Inhibition of IFN signaling by NiV V protein has been demonstrated in African green monkey Vero cells, two pig cell lines (PK-15 and PKIBRS2), MDCK dog cells, RK rabbit cells, NBL-6 horse cells, and Tb1 Lu cells derived from the insectivorous bat *Tadarida brasiliensis* [59]. These findings align with the ability of NiV to replicate in diverse species, including humans, pigs, dogs, and horses [7]. Similarly, Mapuera virus (MPRV), another paramyxovirus, encodes a V protein that inhibits ISRE activation in HeLa cells [60]. It was isolated from a fruit bat, but its host range remains unknown [61]. *Co-*IP experiments and microscopic analysis performed in human Hep2 cells revealed that MPRV V protein interacts with both STAT1 and STAT2, blocking their nuclear translocation [60]. MPRV V also inhibited the induction of an ISRE-responsive promoter in Tb1-Lu lung epithelial cells from *T. brasiliensis*, as well as in monkey (Vero), dog (MDCK), horse (NBL-6), and porcine (PK-15, PKIBRS2) cells, but not in murine BF cells [60]. Thus, the ability of MPRV and NiV V protein to counteract IFN responses, through an interaction with STAT1/2, across multiple mammalian cell types is a critical determinant of their host range and pathogenicity.

Severe fever with thrombocytopenia syndrome (SFTS) is an emerging human disease caused by the SFTS virus (SFTSV), which belongs to the *Bunyaviridae* family [62]. The expression of the viral NS protein inhibits the activation of ISRE in human 293T cells, but not in mouse NIH 3T3 cells [63]. Consistently, co-IP assays revealed that NS interacts with hSTAT2 but not mSTAT2 [63]. The inability of SFTSV NS to interfere with IFN signaling in murine cells could explain the lack of pathogenicity of SFTSV in mice.

Similar to SFTSV NS, the anti-STAT2 activity of three human orthoflaviviruses (DENV, ZIKV, and yellow fever virus [YFV]) contributes to species-specificity of infection. The NS5 protein of these three related mosquito-borne orthoflaviviruses interacts with hSTAT2 [64–68]. Infection with YFV inhibits ISG expression in 293T cells but not in murine Hepa1.6 cells following IFN-I treatment [69]. Co-immunoprecipitation assays on IFN-I-treated STAT2-deficient U6A human cells co-transfected with YFV NS5 and either mSTAT2 or hSTAT2 showed that the binding of YFV NS5 was specific to hSTAT2 [69]. This binding requires an IFN-I-dependent ubiquitination of NS5 that does not occur in mouse cells [69]. Infection with ZIKV of primary human and mouse fibroblasts revealed that STAT2 levels were reduced in human cells, but not in mouse cells [66]. Similarly, **Stat2*^*−/−*^* mouse fibroblasts and U6A cells transfected with mSTAT2 or hSTAT2 and infected with DENV resulted in the loss of expression of hSTAT2, but not mSTAT2, in both cell lines [70]. Consistently, co-IP assays performed in 293T cells transfected with ZIKV NS5 or in hamster BHK-21 cells transfected with DENV NS5, together with either human or mouse STAT2, demonstrated that both NS5 could bind and degrade hSTAT2 but not mSTAT2 [66,70]. The resistance of mSTAT2 to NS5 has implications for *in vivo* models, as only immunocompromised or humanized STAT2 mice are susceptible to orthoflavivirus infection. STAT2 resistance has evolved independently at least twice in rodents and similar resistance is observed in other mammals, such as lemurs and bats [71]. In these resistant species, multiple amino acid substitutions emerged in the NS5-binding residues of STAT2 without compromising its signaling function, illustrating how orthoflavivirus IFN antagonism has shaped STAT2 evolution across mammals [71].

### Inhibiting the activities of IFN effectors by viral proteins can be host-specific

A study comparing IFN-induced transcriptomic changes in primary dermal fibroblasts across nine mammalian species identified a core set of 90 conserved ISGs (Fig 1) [72]. These ISGs play key roles in antiviral defense, antigen presentation, protein degradation, cell signaling, and apoptosis and include well-known antiviral effectors, such as Viperin, MX1, IFITM3, and OAS1. The study also uncovered species-specific ISG signatures, with certain ISGs detected exclusively in individual species. This suggests that lineage-specific ISGs are more widespread than previously thought and may have arisen through exposure to different viruses [72].

#### APOBEC3.

The members of the apolipoprotein B mRNA-editing enzyme, catalytic polypeptide-like3 (APOBEC3/A3) family of enzymes are IFN-induced cytosine deaminases that target ssDNA or RNA structures [73]. In primates, this family includes seven members (A3A, A3B, A3C, A3D, A3F, A3G, and A3H), some of which exhibit potent antiviral activity against lentiviruses [73]. While not all A3 proteins restrict lentiviral replication, those that do localize in the cytoplasm and are incorporated into nascent virions through interactions with viral genomic RNA or proteins [73]. These encapsidated A3s inhibit viral replication in newly infected cells via two mechanisms: physical obstruction of the reverse transcription of the viral RNA genome [74] and hypermutation of the viral genome through cytosine deamination [75–77].

However, the Vif proteins of multiple human and simian lentiviruses, including HIV-1, HIV-2, and SIV [73], mediate species-specific degradation of A3 protein via the proteasome. For instance, HIV-1 Vif degrades chimpanzee and human A3G orthologs, but not those from rhesus macaques, African green monkeys (AGMs), or mice [78–80]. By contrast, Vif from a simian lentivirus (SIV_agm_), targets AGM A3G but not human A3G [79]. Mutational and structural studies have identified amino acids 128–130 as a critical determinant of this species-specific sensitivity [81]. Notably, substituting aspartate 128 with a lysine in human A3G (hA3G), mimicking AGM A3G, prevents HIV-1 Vif binding [80], thereby conferring resistance to Vif-mediated degradation and restoring antiviral function.

A3F also contributes to lentiviral CST barriers. Different HIV-2 lineages emerged from SIVs circulating in sooty mangabeys (SIV_smm_) through several spillover events [82]. The production of infectious SIV_smm_ particles, but not of HIV-2, was diminished by hA3F in 293T cells [82]. Consistently, hA3F is more effectively degraded by HIV-2 replication than SIV_smm_ [82]. Overexpression of monkey A3F variants, on the other hand, did not influence SIV_smm_ production in 293T cells. A mutation at position 128 (R128T in human and T128R in smm) revealed the importance of this amino acid for the functionality against lentiviral infection [82]. While the R128T change in human A3F increased the antiviral activity of A3F against HIV-1 and HIV-2, the reverse mutation increased the potency of smA3F against SIV_smm_.

Finally, a hA3H variant resists degradation by Vif from chimpanzee- and gorilla-derived SIVs, but remain sensitive to HIV-1 [83]. In contrast, chimpanzee A3H is antagonized by both SIV and HIV-1 Vifs. Only two amino acid substitutions in the chimpanzee SIV Vif (E47N/P48H) were sufficient to trigger the activation of proteasomal degradation of hA3H. These examples underscore how minimal sequence variations in viral proteins can significantly influence interspecies transmission dynamics [83].

#### BST-2.

BST-2 also known as CD317 or tetherin. This ISG product inhibits the release of diverse enveloped viruses that assemble at the cellular surface, including retroviruses and filoviruses [84–86]. The HIV-1 Vpu protein interferes with the transport of tetherin to the cellular surface, thereby preventing its antiviral activity [87]. Notably, HIV-1 Vpu antagonizes human and chimpanzee tetherin but not orthologs from other primate species [88]. Conversely, Vpu proteins from simian lentiviruses, like SIV_cpz_ and SIV_gor_, cannot counteract human tetherin, or orthologs from their natural host species [89]. Instead, these SIVs, along with other SIVs infecting Old World monkeys, antagonize tetherin with their Nef proteins [89–91]. Positive selection analyses showed that the tetherin residues interacting with Old World monkey Nef are located in a region deleted in human tetherin [89], explaining why human tetherin is insensitive to Nef. The acquisition of anti-tetherin function by Vpu thus compensated for Nef’s inability to target human tetherin [89].

Ebola virus (EBOV) is a highly pathogenic filovirus in humans. EBOV-infected cells secrete microvesicles containing the viral glycoprotein (GP). These microvesicles act as decoys for EBOV neutralizing antibodies [92]. The formation of these microvesicles is restricted by tetherin from various species, including human, hamster, alligator, and fruit bats (*Epomops buettikoferi* and *Hypsignathus monstrosus).* The fruit bat species are of particular interest as they may constitute the natural reservoir for EBOV [93]. In human 293T cells co-expressing EBOV GP and tetherin from various species, tetherin from the 2 fruit bats was the most efficient at blocking the formation of the microvesicles [92]. This suggests an efficient control of EBOV replication in cells from the natural hosts that is not available in human cells, potentially contributing to the heightened pathogenicity observed in human infections.

#### CNP.

2’,3’-cyclic-nucleotide 3’-phosphodiesterase (CNP) is another ISG with antiretroviral functions. CNP is a membrane-associated enzyme that restricts lentiviral replication by blocking the virion assembly through binding to the structural viral Gag protein at the plasma membrane [94]. CNP orthologs from human, macaque, AGM, cow, and sheep, but not murine CNP, potently restrict the assembly of a subset of lentiviruses in 293T cells [94]. Mutational experiments with chimeric CNP proteins revealed that the inhibitory potential of hCNP can be abrogated by a single amino acid change at position 72 (D72E), which is present in mCNP [94]. Importantly, the reverse mutation in murine CNP (E72D) did not lead to antiviral activity, implying that more than one residue is causing the divergent phenotypes [94].

#### IFIT1.

Human interferon-induced protein with tetratricopeptide repeats 1 (IFIT1) is a broad-spectrum antiviral factor [95] that suppresses viral RNA translation through its RNA-binding properties [96]. A comprehensive screen performed in human cells expressing IFIT1 orthologs from 39 mammalian species revealed species-specific antiviral activity against several RNA viruses [96]. For instance, human and bat IFIT1, but not the chimpanzee ortholog, exhibited antiviral activities against Venezuelan equine encephalitis virus (VEEV), an alphavirus transmitted by mosquitoes [96]. Mutagenesis studies comparing IFIT1 variants with differential anti-VEEV activity identified two critical amino acids that determine this functional difference. These residues are not directly involved in RNA-binding but may be involved in conformational changes of IFIT1 during RNA-binding [96].

#### ISG15.

ISGylation is a posttranslational modification consisting of the conjugation of ISG15 to proteins [97]. ISG15 orthologs across different mammalian species are widely diverse and share as little as 60% sequence identity [98].

Nairoviruses are predominantly tick-associated pathogens with global health significance. They encode for a homologue of the ovarian tumor domain protease (OTU) that can reverse ISG15-mediated posttranslational changes. Testing ISG15 orthologs from 11 mammalian species against 14 nairovirus OTUs revealed that six viral proteins (originating from Kupe virus [KUPEV], Dugbe virus [DUGV], Nairobi Sheep Disease virus [NSDV], Ganjam virus [GANV], Crimean-Congo hemorrhagic fever virus [CCHFV], and Erve virus [ERVEV]) showed a broad ISG15 cleavage activity across multiple species [98]. All OTUs with cleaving activity processed bovine and ovine ISG15. Only NSDV, GANV, CCHFV, and ERVEV OTUs targeted human ISG15 [98]. Finally, none of the OTUs could cleave Egyptian fruit bat (*Rousettus aegyptiacus*) ISG15 [98]. Structural and mutational analyses identified seven critical residues in ISG15 (positions 89, 130, and 149–151) that determine species-specific OTU-ISG15 interactions [98].

Similar studies with coronavirus papain-like proteases (PLPs) revealed species-specific activity patterns against mammalian ISG15 orthologs interaction [99]. Structural and biochemical analysis identified a unique twisted hinge region in ISG15, absent in the human ortholog, as crucial for these differences in PLP-ISG15 interaction [99].

Influenza B viruses (IBV) are human viruses that inhibit ISGylation through their NS1 proteins in human cells. Co-immunoprecipitation experiments in 293T cells showed that IBV NS1 binds to and inhibits human, mouse, canine, and African green monkey ISG15, but not murine or canine orthologs [100]. These results may explain why IBV can replicate in humans but not in mice.

#### Mx1/Mx2.

Myxovirus resistance (Mx) proteins are a family of IFN-induced dynamin-like GTPases first identified for their ability to confer resistance to lethal doses of IAV in mice [101]. Most mammals encode two paralogous Mx proteins, Mx1 and Mx2. The human paralogs, which arose from an ancient duplication event, are referred to as MxA and MxB. MxA protein targets the nucleoprotein (NP) of Thogoto virus (THOV), an influenza-like *orthomyxovirus* transmitted by ticks, to inhibit viral replication [102]. An evolution-guided functional analysis across 24 primate species, representing over 40 million years of evolutionary divergence, identified genomic signatures determining MxA’s virus specificity [103]. This analysis revealed that the unstructured L4 loop in the MxA stalk domain is significantly enriched in positively selected sites. Monitoring the replication of a THOV minireplicon in the presence of 15 diverse primate MxA orthologs showed a reduced antiviral activity of great ape orthologs and a complete loss of antiviral function in MxA proteins from gibbons, New, and Old World monkeys [103]. Comparative analysis of chimera MxA proteins with and without antiviral activities pinpointed a single amino acid change at position 561 in the L4 loop as critical for THOV restriction [103]. Subsequent mutational experiments confirmed this residue’s importance, as swapping the human and AGM residues resulted in a complete reversal of antiviral phenotypes against IAV in 293T cells. While bat-derived Mx1 orthologs lack antiviral activity against THOV when expressed in 293T cells, Mx1 from *Carollia perspicillata*, *Pipistrellus pipistrellus*, and *Eidolon helvum* showed antiviral activity against multiple orthomyxoviruses (H5N1, H7N7, H17N10) in the same cells [104]. Additional experiments in 293T cells using VLP assays and a minireplicon system identified that both human and bat Mx1 orthologs inhibit the replication of VSV (*Rhabdoviridae*), EBOV (*Filoviridae*), and RVFV (*Bunyaviridae*) [104]. This highlights the broadly functional and evolutionary conserved antiviral properties of Mx1 across mammalian species. Notably, in all cases examined, the ability of Mx1 to counteract viral replication could be abolished by a single amino acid substitution, highlighting both the flexibility and fragility of this host defense strategy [104]. The inability of bat-derived Mx1 proteins to restrict THOV replication, in contrast to their human ortholog, remains unexplained. This observation suggests that the examined bat species may not have encountered THOV in their evolutionary history and consequently have not developed antiviral strategies against this virus.

MxB restricts the replication of HIV-1 and herpesviruses [105–107]. It inhibits HIV-1 infection prior to nuclear import of viral complexes, but after completion of reverse transcription [105,106]. The antiviral mechanism of MxB involves recognition of the viral capsid protein (CA), as demonstrated by the fact that single amino acid mutations in the CA can confer viral escape from MxB restriction [108]. Human, macaque, and AGM MxB orthologs restrict HIV-1 replication, while ovine and canine MxB proteins show no antiviral activity in human MT-4 cells [108]. Notably, the exchange of a small region of canine MxB with its human Mx2 counterpart was sufficient to confer anti-HIV-1 activity, indicating that canine MxB requires minimal changes to gain antiviral function [108]. The antiviral specificity of Mx2 extends to different HIV-1 variants: M-group HIV-1 is restricted by human, macaque, and AGM MxB while O-group HIV-1 is restricted only by human Mx2. This species-specific restriction was mapped to two residues at positions 37 and 39 at the N-terminus of Mx2 proteins, as demonstrated using chimeras of human and AGM proteins [108]. Recent studies have expanded our understanding of MxB’s antiviral spectrum. Human Mx2 protein restricts HSV-1 replication in IMR90 cells, whereas most primate orthologs, including closely related chimpanzee MxB, lack this antiviral activity [107]. This species-specific restriction was traced to a single mutation at position 83, as shown by human-chimpanzee MxB chimeric proteins [107]. However, the mechanism by which this single residue governs antiviral specificity remains elusive.

#### OAS.

2′-5′-oligoadenylate synthetases (OASs) are ISGs that encode enzymes activated by dsRNA. This activation leads to the synthesis of 2′,5′-oligonadenylates (2-5A) that activate RNaseL. RNaseL, in turn, degrades cellular and viral RNA [109]. Theiler’s murine encephalomyelitis virus (TMEV), a murine picornavirus, inhibits the OAS/RNaseL pathway in a species-specific manner. The viral L* protein interacts with the ankyrin domain of RNAse L, preventing its oligomerization and thereby inhibiting its activity in mice [110]. Degradation of RNA was observed in human (HeLa), equine (ED), canine (MDCK), porcine (PK-15), bovine (MDBK), chicken (HD11), hamster (CHO-K1), but not in murine (L929), nor rat (AR42J) cells, expressing the L* protein [110]. This species-specific pattern of RNase L inhibition correlates with the ability of TMEV and related viruses to establish productive infections in murine and rat cells, suggesting an evolutionary adaptation to these natural hosts’ antiviral defenses.

An arrayed ISG expression screen performed in human lung A549-AT cells infected with SARS-CoV-2 identified human OAS1 as a potent antiviral factor against the virus [111]. This antiviral activity depends on a C-terminal prenylation of hOAS1, which enables the protein to localize to perinuclear replication organelles where viral dsRNAs accumulate. Interestingly, this antiviral mechanism shows virus specificity: hOAS1 does not affect OC43 replication, regardless of its prenylation status. This differential sensitivity to hOAS1 can be explained by viral countermeasures: several coronaviruses, including OC43 and MERS-CoV, encode phosphodiesterases (PDEs) to counteract the hOAS/RNAseL system. The evolutionary pressure to express these PDEs likely originate from the presence of prenylated OAS1 in intermediate hosts (mice and cows for OC43, bats and camels for MERS-CoV) [111]. In contrast to hOAS1, OAS1 orthologs in bats of the Rhinolophoida superfamily, which are believed to include the species from which SARS-CoV-2 originated [112], lack a prenylation site. Consequently, OAS1 isoforms from the greater horseshoe bat (*Rhinolophus ferrumequinum*) are unable to block SARS-CoV- 2 replication in human A549 cells modified to be permissive for the virus [111]. However, OAS1 derived from *P. Alecto*, a megabat with intact OAS1 prenylation, effectively inhibits SARS-CoV-2 replication in the same cells [111]. These findings suggest that prenylated OAS1-mediated dsRNA sensing may be absent in potential natural bat reservoirs of SARS-CoV-2. The lack of pressure to evade prenylated OAS1 in these bat species may have resulted in SARS-CoV-2 being sensitive to hOAS1. This example illustrates how evolutionary changes in mammalian genomes resulted in diverse repertoires of antiviral defenses that influence CST of viruses.

#### PHF11.

A screening approach performed with a cDNA library of rhesus macaque ISGs recovered plant homeodomain finger protein 11 (PHF11) as a potent restriction factor against prototype foamy virus (PFV) in human HT1080 cells [113]. PFV belongs to the family of foamy viruses, an ancient subfamily of retroviruses that are primarily endemic in nonhuman primates but show a broad tropism across mammalian species [114]. Foamy viruses are apathogenic*,* suggesting effective control by their host defense mechanism. Macaque PHF11 plays a crucial role in host control by interfering with gene expression from an internal viral promoter, a replication feature unique to foamy viruses. However, this antiviral activity shows remarkable species specificity. It was only observed with PHF11 derived from macaques and humans when tested against several foamy viruses in human epithelial-like cells expressing GFP (U3-GFP) or feline epithelial-like (CRFK) cells [115]. In contrast, overexpression of murine and feline PHF11 orthologs failed to significantly restrict viral replication in the same cellular models [115]. Structural and functional analyses using PHF11 protein chimeras, where N-terminal, C-terminal, and PH domains were exchanged, revealed that all three domains contribute to the antiviral activity of human PHF11. This suggests that multiple sequence alterations are responsible for the loss of antiviral activity in feline and murine PHF11 orthologs [115]. This hypothesis is supported by sequence comparisons showing that orthologs of macaque and human PHF11 share 94% identical amino acid sequence, while feline (61%) and murine (42–50%) orthologs exhibit greater divergence [115].

#### RTP4.

A gain-of-function screening approach conducted in kidney PaKi cells derived from the black flying fox (*Pteropus alecto*) identified receptor transporting protein 4 (RTP4) as a potent IFN-inducible antiviral effector against the orthoflaviviruses ZIKV and DENV [116]. The antiviral mechanism of *P. alecto* RTP4 (paRTP4) involves direct binding to viral double-stranded RNA produced during replication, significantly reducing viral RNA synthesis [116]. Both paRTP4 and its human ortholog (hRTP4) restricted the replication of a panel of orthoflaviviruses. Moreover, paRTPT4 also restricted the replication of HCV, which belongs to another genus of the *Flaviviridae* family, as well as two unrelated viruses, equine arteritis virus, and human coronavirus OC43. Comparative studies using RTP4 orthologs from nine different mammalian species expressed in human Huh7.5 cells revealed a pattern of species-specific viral resistance, where viruses showed the greatest resistance to RTP4 from their natural hosts—for example, HCV and YFV to hRTP4, and Entebbe bat virus (ENTV), a bat-borne orthoflavivirus with no known arthropod vector, to paRTP4. Serial passaging of YFV and ENTV in RTP4-overexpressing cells demonstrated their ability to rapidly develop evasion strategies against antiviral RTP4 orthologs from rhesus macaque or Mexican free-tailed bat. These findings collectively suggest that the ability of orthoflaviviruses to antagonize RTP4 plays a role in determining their host range.

#### TRIM5α.

Tripartite motif 5α (TRIM5α) was among the first ISGs recognized for its crucial role as a barrier to cross-species viral transmission [117–119]. This antiviral factor employs multiple mechanisms to block the replication across diverse viral families, though it has been extensively characterized for its activity against retroviruses [120]. TRIM5α variants from humans, rhesus macaques, and AGMs effectively restricted retroviruses circulating in other primate species and equines, they showed diminished capacity to inhibit retroviruses isolated from their own species when overexpressed in feline CRFK cells [117]. Subsequent comparative analyses of humans and rhesus macaques TRIM5α orthologs regarding their potential to block HIV-1 or SIV replication uncovered that these differences in antiviral potential stem from sequence variation in the N-terminal region of the SPRY domain of TRIM5α [118,119]. Mutational studies revealed that only one to three amino acid substitutions in this domain were sufficient to restore TRIM5α ability to restrict replication of retroviruses isolated from the same species, underlining the fragility of these species-species barriers [119].

#### TRIM69.

Using a lentivirus-based ISG expression screening approach in human MT4 cells, TRIM69 was identified as a potent human ISG against vesicular stomatitis Indiana virus (VSIV) [121]. While VSIV typically causes mild infections in humans, it can induce severe disease in ungulates. Although the mechanism behind the antiviral potential of TRIM69 against VSIV remains elusive, overexpression experiments in human MT4 cells revealed species-specific differences in antiviral potency. Specifically, TRIM69 orthologs from human, rhesus macaque, rat, cow, alpaca, pig, dog, ferret, and horse diminished VSIV titers, whereas expression of three different murine TRIM69 orthologs (from *Mus musculus*, *M. caroli*, and *M. pahari*) did not affect viral replication in MT4 cells [121]. While the molecular basis for these divergent phenotypes was not investigated, these findings provide yet another example of sequence variations in ISGs that can influence viral species tropism.

#### Viperin.

The human RSAD2 gene encodes for viperin, an ISG product that exhibits antiviral activity against diverse RNA and DNA viruses [122]. This evolutionarily conserved protein employs multiple mechanisms to restrict viral replication across mammalian species [122]. As with many antiviral factors, viruses also evolved strategies to evade the antiviral effects of viperin. For instance, HSV-1 encodes for UL41, an endoribonuclease, which interferes with viperin function by reducing RSAD2 mRNA levels [123]. Examination of the antiviral potential of overexpressed FLAG-tagged human and murine viperin in HSV-1-infected 293 cells revealed that only murine viperin diminished viral replication [124]. Monitoring viperin expression in a panel of human (293A, HeLa, HFF, and MRC5) and murine (MF) cells showed that HSV-1 infection triggered RSAD2 upregulation only in murine cells in the first 8 hours of infection [124]. These findings suggest that HSV-1 has evolved a host-specific evasion strategy capable of counteracting human viperin expression while remaining susceptible to viperin from other mammalian species, potentially representing an important barrier to interspecies transmission.

#### 90K.

The ISG gene product galectin 3 binding protein (LGALS3BP, also known as 90K) represents another potent antiviral factor with activity against retroviruses [125]. This protein restricts HIV-1 infectivity by limiting Env incorporation into nascent virions [125]. 293T cells, which lack endogenous 90K expression, were modified to express either human 90K or one of seven nonhuman primate orthologs [125]. Analysis of viral particles released from these cells revealed that 90K proteins from humans, chimpanzees, orangutans, AGMs, gibbons, baboons, and owl monkeys all similarly restricted the infectivity of HIV-1, SIV_mac_, and SIV_smm_. Only the rhesus macaque 90K failed to exhibit antiviral activity [125]. However, all tested 90K proteins were active against HIV-2, SIV_agm_, and SIV_cpz_. Structural and functional analyses of human and macaque 90K proteins identified a critical single amino acid difference at position 142 that accounted for the macaque protein’s inactivity against HIV-1. Humanizing this single residue in the macaque protein fully restored its antiviral activity against HIV-1, demonstrating how minimal sequence variations can impact antiviral specificity [125].

**Fig 2:**
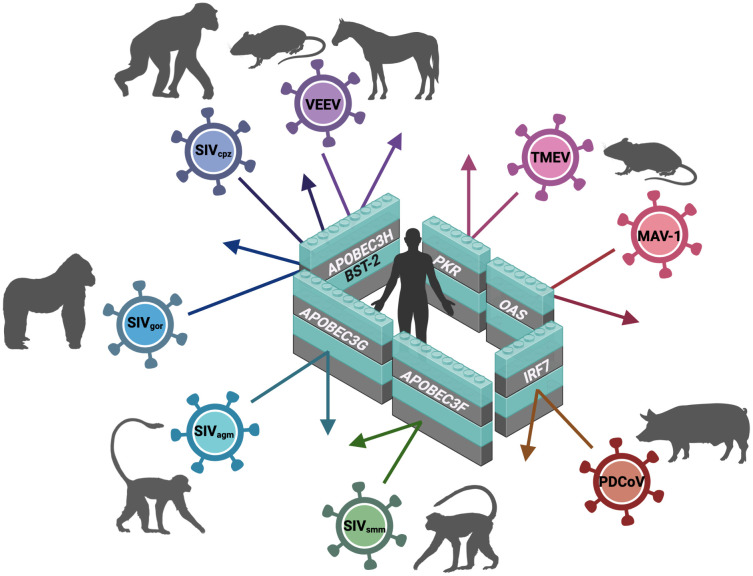
Simplified overview of molecular interspecific barriers for animal-borne viruses restricting transmission to humans. Simian immunodeficiency (SIV)_cpz_ is hosted by *Pan troglodytes* (chimpanzee); Venezuelan equine encephalitis virus (VEEV) is hosted by rodents and horses; Theiler’s murine encephalomyelitis virus (TMEV) and mouse adenovirus type 1 (MAV-1) are hosted by *Mus musculus*; porcine deltacoronavirus (PDCoV) is hosted by *Sus scrofa domesticus*; SIV_smm_ is hosted by *Cercocebus atys* (sooty mangabey monkey); SIV_agm_ is hosted by *Chlorocebus aethiops* (African Green monkey) and SIV_gor_ is hosted by *Gorilla gorilla*. Abbreviations: APOBEC3F/G/H, apolipoprotein B mRNA editing enzyme catalytic subunit 3F/G; BST-2, bone marrow stromal cell antigen 2; PKR, protein kinase R; OAS, 2’-5’-oligoadenylate synthetase; IRF7, interferon regulatory factor *7.* Created in BioRender. https://urldefense.com/v3/__https://BioRender.com/0a8cb2c__;!!JFdNOqOXpB6UZW0!qE_dfBruIlmSU-rmo3ya4I37cy0eQrZ8nhz6vRSpTfifiY_79LhLogYEkPhs4-N9D11wwYm62NAE8nJ7CU4fYsf1NQ$.

These studies collectively show how the evolutionary arms race between viruses and their mammalian hosts has shaped the evolution of ISGs, frequently resulting in species-specific antiviral effects (Table 1, Fig 2). They further underscore that even minimal changes in the sequences of viral or host effectors can significantly alter virus-host interactions, thereby facilitating CST of viruses.

**Table 1:** Overview of molecular interspecific barriers for human pathogens towards other mammalian species.

Virus	Barrier	Protected species	Ref.
MAVS/RAVV	Jak1	*Mus musculus*	[53]
HIV-1	APOBEC3G	*M. musculus, Chlorocebus aethiops, Macaca mulatta*	[78–80]
	BST-2	*M. musculus, C. aethiops, Ma. mulatta*	[88,90]
	MxB	*Ovis aries, Canis lupus*	[108]
	TRIM5α	*Ma. Mulatta, C. aethiops*	[117,119]
HIV-2	APOBEC3F	*Cercocebus atys*	[82]
IAV	TRIM25	*M. musculus*	[18]
IBV	ISG15	*M. musculus*	[100]
HSV-1	RIG-I	*M. musculus*	[14]
	cGAS	*Lophocebus aterrimus, Erythrocebus patas, Ma. mulatta, Pongo abelii*	[26]
	RSAD2	*M. musculus*	[124]
HAV	MAVS	*M. musculus*	[23]
HCV	MAVS	*Papio anubis, Ma. mulatta*, *Plecturocebus moloch, Ateles geoffroyi*	[22]
	RTP4	*M. musculus, Sus scrofa domesticus, Bos taurus, Tadarida brasiliensis, Rousettus aegyptiacus, Pteropus alecto*	[116]
DENV	STING1	*M. musculus, Pan troglodytes, Ma. mulatta, Callithrix jacchus*	[27,31]
	STAT2	*M. musculus*	[70]
ZIKV	STING1	*M. musculus, Ma. mulatta, Saimiri sciureus, P. troglodytes*	[126]
	STAT2	*M. musculus*	[127]
YFV	STAT2	*M.musculus*	[69]
HCMV	PKR	*Ma. mulatta, C. aethiops*	[37]
SFTSV	STAT2	*M. musculus*	[128]
VACV	PKR	*Mesocricetus auratus, Me. brandti*	[45]

**Abbreviations:** MAVS, Marburg virus; RAVV, Marburg Ravn virus; HIV-1/2, human immunodeficiency virus type ½; IAV, influenza A virus; IBV. influenza B virus; HSV-1, herpes simplex virus type 1; HAV, hepatitis A virus; HCV, hepatitis C virus; ZIKV, Zika virus; DENV, dengue virus; YFV, yellow fever virus; HCMV, human cytomegalovirus; SFTSV, severe fever with thrombocytopenia virus; VACV, vaccinia virus; Jak1, Janus kinase 1; APOBEC3F/G/H, apolipoprotein B mRNA editing enzyme catalytic subunit 3F/G; BST-2, bone marrow stromal cell antigen 2; MxA/B, MX dynamin like GTPase ½; TRIM5α/25/69, tripartite motif containing 5α/25/69; ISG15, interferon-stimulated protein 15 KDa; RIG-I, retinoic acid-inducible gene I; cGAS, cyclic GMP-AMP synthase; RSAD2, radical S-adenosyl methionine domain containing 2; MAVS, mitochondrial antiviral signaling protein; RTP4, receptor transporter protein 4; STING1, stimulator of interferon response cGAMP interactor 1, STAT2, signal transducer and activator of transcription 2; PKR, protein kinase R.

## Concluding remarks

Viruses have co-evolved with their reservoir hosts over thousands, and sometimes millions, of years. This evolutionary process includes the development of host-specific strategies to evade the IFN response, enabling viruses to spread efficiently within their host tissues. In turn, reservoir host species have evolved mechanisms to tolerate viruses through natural selection, such as reduced inflammatory response. As a result, despite high levels of viremia, reservoir hosts typically exhibit no signs of disease or harm. For example, herpesviruses and their vertebrate hosts have been codiverging for hundreds of millions of years [129]. Specifically, HSV-1 and HSV-2, which exclusively infect humans and do not usually cause harm in healthy individuals, have evolved a large panel of IFN evasion strategies in human cells [130], including human-specific ones, as detailed above. This degree of virus-host coevolution makes herpesviruses poor candidates for CST. Indeed, no evidence for herpesvirus CST between prey and predator species in the primate ecosystem of Western Africa was found, despite considerable physical interactions, genetic similarity, and a high prevalence of herpesviruses in these primates [131].

Adaptation to a new host is facilitated if the virus counteracts the IFN response by targeting conserved cellular proteins across mammalian or even vertebrate species. The closer the genetic relationship between the reservoir host and the new host, the easier the adaptation becomes. For example, SARS-CoV-2 employs a wide array of strategies to evade the IFN response, with most, if not all, of its 29 proteins playing a role [132,133]. This range of IFN antagonistic strategies likely aided the transmission of its ancestor from its reservoir hosts (*Rhinolophus ssp*. bat species) to humans, either directly or through an intermediate host [112,134], and later from humans to other mammalian species (such as white-tailed deer in America, farmed mink in Europe, and tigers and lions in U.S. zoos) [135–139].

Once established in a new host, viruses may also evolve species-specific IFN antagonism strategies. This is facilitated by the error-prone replication of RNA viruses, whose polymerases introduce approximately one mutation per genome per replication cycle, generating high genomic variability and adaptability compared to DNA viruses [140]. For example, SARS-CoV-2 developed reduced sensitivity to human IFNs within months of circulating in humans [141]. The α variant expressed significantly higher levels of Orf9b and Orf6, known potent IFN antagonists, compared to first-wave isolates, enhancing its ability to evade innate immunity [142]. Additionally, it acquired a mutation in its spike protein conferring resistance to the antiviral protein IFITM2 [143], an ISG product known to inhibit SARS-CoV-2 entry [144]. Similarly, pandemic IAV strains have evolved resistance to MxA through mutations in their nucleoprotein (NP) [145]. Although the resistance-associated amino acids differ in their NP between the 1918 and 2009 pandemic strains, they cluster in a discrete, surface-exposed region of NP’s core domain, suggesting that MxA resistance evolved independently during human adaptation [146]. Experimental studies further demonstrate the rapid adaptability of RNA viruses towards the IFN response. For instance, after 6 serial passages of YFV in human cells expressing Mexican free-tailed bat RTP4 (TbRTP4), the virus evolved a mutation in its NS3 protein, enabling it to overcome TbRTP4 antiviral activity [116].

Host genetic variations can also facilitate viral CST. For example, IFITM3 mutations that disrupt correct splicing are linked to increased severity of IAV infections in humans [147]. Consistently, IFITM3-deficient mice and human cells are susceptible to infection with low doses of avian influenza viruses, which fail to infect WT counterparts [148].

To better understand CST, it is crucial to pursue research efforts to identify the innate immune proteins targeted by viruses, as this can provide insights into their host range. If such a protein is conserved across vertebrate species, it will facilitate CST. For instance, the NS5 protein of tick-borne orthoflaviviruses significantly reduces the activation of the JAK-STAT pathway in human cells through a direct interaction with TYK2 [149], a key kinase of the pathway that is well conserved across mammalian species. Experiments with orthologs have revealed that the interaction is conserved across at least 5 mammalian species, including sheep and goats, which are natural hosts of these viruses [149]. These findings suggest that tick-borne orthoflaviviruses may successfully infect a broad range of mammalian species, consistent with their known host range.

Machine learning approaches could enhance our understanding of species-specific viral sensitivity to IFN, thereby aiding in the inference of viral host ranges. If the residues involved in the interactions between cellular and viral proteins are identified, machine learning can predict whether these interactions are conserved across various animal species. For instance, structural and functional studies have pinpointed residues F175 and R176 in hSTAT2 as key targets of the NS5 protein of ZIKV [150]. Machine learning could predict whether the NS5 protein of ZIKV and other mosquito-borne orthoflaviviruses bind to STAT2 orthologs in hundreds of mammalian species, thereby predicting which viruses are resistant to IFN in a given host. Such approaches have recently been employed to predict the susceptibility of bat species to filoviruses based on AI-driven examination of potential interactions between the viral glycoprotein and NCP1, which serves as the viral receptor [151].

Many of the studies cited here rely on the overexpression of individual viral or host proteins. To strengthen these findings, it will be essential to validate them in the context of live-virus infections, ideally using mutant viruses that express proteins unable to interact with their IFN-related targets. Additionally, viral IFN antagonism should be studied in cells derived from the virus’s natural hosts whenever possible. Efforts to develop cellular models, including cell lines, primary cells, and organoids, derived from nonmodel mammalian species, should be made. Recently, organoids were generated from four different organs of five different species of bats belonging to the *Vespertilionidae* and *Rhinolophidae* families. These organoids exhibited an intact IFN response upon infection with MERS-CoV or IAV [152]. These models will facilitate further characterization of the molecular interaction between bat-borne viruses and IFN response in bat cells. In addition to innovative cellular models, other tools to be developed include cDNA libraries of ISG derived from reservoir and amplifying hosts. Transcriptomic analysis of cells stimulated by IFN will be necessary to design these libraries [72]. To date, only human and rhesus macaque ISG cDNA libraries have been generated. Screening these libraries in parallel upon infection with a panel of RNA viruses has revealed differences in the antiviral activities of human and rhesus macaque ISGs [113,115,121,153,154]. Finally, since RNA viruses evolve rapidly in the cells in which they are isolated and produced, primary viral isolates should be studied whenever possible. These models and tools will help predict whether a given virus is a good candidate for CST.

Predicting the emergence of a virus obviously remains a significant challenge, as it results from complex interactions with its host, not only at a molecular level, but also influenced by broader factors. Human-induced changes in ecosystems, urbanization, global trade, migration, and climate change all play a crucial role. Effective prevention and control of zoonotic diseases thus require collaborative efforts across multiple disciplines, including human health, animal health, epidemiology, environmental science, politics, and economics. These efforts should particularly focus on regions where human-wildlife and livestock interactions are significant [155].
